# On the Mechanism of Speech

**Published:** 1865-04

**Authors:** Isaac Pidduck


### ON THE MECHANISM OF SPEECH.

By ISAAC PIDDUCK, M. D.

To compare the mechanism of speech to that of a musical instrument, the organ, for instance: the chest is the bellows; the abdomen is the blower; the throat is the windpipe; the larynx is the reed; and, besides these, the cartilages and vocal cords are the strings; showing that the sounds of the voice are produced by the combination of a wind and a stringed instrument.

The sound formed by the larynx, (consisting of the rima or chink,) the cartilages, and the vocal cords, is divided by the tongue, the palate, the cheek, the teeth, and the lips, into letters, syllables, words, and sentences. Upon the perfect formation and healthy condition of these several parts, the strength, the rhythm, and the distinctness of the voice depend.

But to play skillfully on a musical instrument, long and careful practice is required. All persons learn to speak, as some persons learn to sing, by the ear; but very few either speak or sing correctly unless the organs of voice have been properly taught, and brought into perfect subjection by regular instruction and strict discipline. It was related of three young ladies, sisters, that they could sing, but not read aloud for any length of time, without becoming hoarse and losing their voice. They had been taught to sing, but not to speak.

By way of illustration, let us consider—

1st. The chest, or bellows. In order to speak with a loud and clear voice, so as to be heard distinctly at a distance, the capacity of the chest should be ample. This may be increased by exercise, by taking deep inspirations, and by holding the breath a short time when the chest is full, suffering the air to escape gradually by counting aloud 1, 2, 3, etc., up to 50 or 70. By this means the chest may be expanded in every direction, and its capacity greatly enlarged.

2d. The abdomen, or blower. Fullness of the abdomen is caused by eating and drinking largely, and this, again, causes flatulence, distension, or the deposition of fat, especially by the neglect of exercise; and this impedes the muscular action of the abdomen in expelling the air from the chest with sufficient force to produce a full volume of voice.

3d. The trachea, or windpipe. And 4th. The larynx. These most important parts are liable to catarrhal and nervous affections by which the voice is lost, or is rendered hoarse and discordant—*vox faucibus hæsit.* The causes which produce this injurious effect are—breathing a close, heated atmosphere, wearing warm wraps round the throat, drinking freely of hot liquids, particularly hot tea, spirit-drinking, snuff-taking and tobacco-smoking, the use of voice-lozenges, straining the voice beyond its compass, and by frequently clearing the throat. The articulate sounds, the rhythm and *timbre* of the voice, being formed by the larynx, compounded of the cartilages, the rima, or chink, and the vocal cords, and being divided by tongue applied to the palate, the teeth, and the lips into letters, syllables, words, and sentences, it is most important that all these several parts should be in a normal and healthy condition. If the tongue be too large or too small; if the palate be cleft, too arched, or too flat; if the cheeks or the lips be too largely or not fully developed, and the teeth be defective, the voice will be deficient in power, and the words will be indistinctly pronounced.

In order to attain an effective elocution, the following rules should be observed:—

1st. The speaker should stand erect, and the head not bent upon the chest, that the muscular movements of the abdomen, chest, and throat may be free and unconstrained.

2d. The chest should be fully expanded by each inspiration at the commencement of every sentence. The disregard of this rule is a frequent cause of stammering. To fill the chest and to hold out the breath to complete each sentence, the inspiration should be made through the nose. By this mode of inspiring though the nostrils, the mouth and throat are prevented from becoming dry and the voice from becoming hoarse.

3d. The pauses should be long enough for each sentence to reach its destination before it is followed by another; and, *cæteris paribus*, the slowness of the utterance should be in the ratio of the size of the room and the number of the audience.

"Learn to speak slow: all other graces,
Will follow in their proper places."

4th. Every word, if not every syllable, and almost every letter, should be distinctly enunciated, that the attention of the auditory may not be diverted from the sense to catch the sound. By this two-fold effort the attention soon grows weary and the hearer listless, and then instruction or amusement ceases.

Among the faults of extemporary speakers, lecturers, and preachers, rapidity of utterance is one of the most common. Deliberation gives time for the choice of words; and, in consequence, the speech, the lecture, or sermon is more effective, is less tedious to the hearers, and commands greater and longer attention. This rule requires self-possession, a perfect knowledge of the subject, and an earnest desire on the part of the speaker to enlighten and instruct his auditory. Rapidity of reference and of quotation may excite astonishment, but it does not impart information, which should descend upon the mind as the dew from heaven.—*Lancet.*

